# Imaging characteristics of pulmonary BCG/TB infection in patients with chronic granulomatous disease

**DOI:** 10.1038/s41598-022-16021-9

**Published:** 2022-07-11

**Authors:** Qiong Yao, Qin-hua Zhou, Quan-li Shen, Xiao-chuan Wang, Xi-hong Hu

**Affiliations:** 1 Department of Radiology, Children’s Hospital of Fudan University, Shanghai, 201102 China; 2 Department of Allergy and Clinical Immunology, Children’s Hospital of Fudan University, Shanghai, 201102 China; 3 Cardiac Center, Children’s Hospital of Fudan University, Shanghai, 201102 China

**Keywords:** Immunology, Diseases

## Abstract

In China, tuberculosis (TB) is endemic and the Bacillus Callmette–Güerin (BCG) vaccine is administered to all the newborns, which may lead to BCG infection in patients with chronic granulomatous disease (CGD). Infection of BCG/TB in CGD patients can be fatal and pulmonary is the most affected organ. Our objective was to assess the imaging of pulmonary BCG/TB infection in CGD. We screened 169 CGD patients and identified the patients with pulmonary BCG/TB infection. BCG infection was diagnosis according to the vaccination history, local infection manifestation, acid-fast bacilli staining, specific polymerase chain reaction, and/or spoligotyping. PPD, T-SPOT and acid-fast bacilli staining were used for diagnosis of TB. Totally 58 patients were identified, including TB (n = 7), solely BCG (n = 18), BCG + bacterial (n = 20), and BCG + fungi (n = 13). The onset of BCG disease was much earlier than TB. For those patients only with BCG, lymphadenopathy was the first and most prevalent feature. The most found location was the left axilla, followed by the ipsilateral cervical areas and mediastinal or hilar area. On chest CT, ground-glass opacities, multiple nodules and pulmonary scarring were the most common findings. For TB patients, the pulmonary infections were more serious, including large masses, severe lymphadenopathy, and extensive pulmonary fibrosis. Pulmonary infection of BCG were more common than TB in CGD patients, but much less severe.

## Introduction

Chronic granulomatous disease (CGD) is a primary immunodeficiency disease (PID) characterized by the deficiency of nicotinamide adenine dinucleotide phosphate (NADPH) oxidase to produce reactive oxygen species. Five types are the most common types according to the mutation of genes: gp91phox (CYBB) and p22phox (CYBA), the cytosolic subunits p47phox (NCF1), p67phox (NCF2), and p40phox (NCF4)^1,2^.

Patients usually experience recurrent infections caused by a relatively specific set of catalase positive pathogens, mainly bacterial and fungal infections (Aspergillus, Burkholderia, Nocardia, and Staphylococcus species). In China, tuberculosis (TB) is endemic and lung is the most affected organ^3^. In 2015, the incidence of pulmonary TB in children (0–14 years old) of China was 3.03 per 100,000^4^. To prevent severe TB infection, Bacillus Callmette–Güerin (BCG) vaccination is routinely administered to every newborn. It is one of the most widely used vaccines in over 150 countries and proved to be safe in immunocompetent hosts^5,6^. However, for PID patients including CGD, it will lead to BCG infection^7^. Most present with local BCG disease, mainly regional lymphadenitis^5,6^. Disseminated BCG with CGD is extremely rare but fatal with a mortality rate of 60–80%^8,9^. So in China, in addition to the commonly pathogens, BCG and TB infection should also be considered in the clinical work^2,10–12^.

We have performed retrospective reviews on the clinical manifestation of CGD patients and identified the high incidence of BCG infection, which can also be found in other countries in Asia, South Africa and Latin America^2,10,11^. Patients with BCG and TB infection all present positive in PPD tests and prolonged and recurrent pulmonary infection, which sometimes hard to be distinguished in the clinical work. Chest CT is the routine exam for GCD patients and can afford useful information for identifying the pathogens^13^. However, for pulmonary BCG/TB infection, researches focusing on imaging findings are limited due to the low morbidity. This is the first research to summarize the imaging manifestations of pulmonary mycobacterial infection and compare the severity between BCG and TB.

## Methods

### Subjects

Our institution is a university teaching hospital, and pediatric Allergy and Clinical Immunology department is a tertiary referral center for patients with PID. From 1999 to 2021, we screened for pulmonary mycobacterial infection in 169 CGD patients with chest CT. The clinical and imaging data were reviewed. Informed consent forms were signed by the parents and ethics approval was approved by the institutional review board of the institution. All methods were performed in accordance with the relevant guidelines and regulations. CGD was diagnosed as described before^2,11^. The defective respiratory burst was detected by dihydrorhodamine-1,2,3 (DHR) test. Decreased protein level of gp91 was tested by flow cytometry-based extracellular staining with Moab 7D5, and gene mutations were identified by Sanger sequencing, including *CYBB*, *CYBA*, *NCF1*, and *NCF2*. Our analysis focused mainly on the imaging manifestations of pulmonary mycobacterial infections. The accompanied infection of other organs was also evaluated. Mycobacterial infections were diagnosed according to the published papers^2,11,14^. BCG infection was diagnosis according to the vaccination history, local infection manifestation, and evidence of acid-fast bacilli staining from pathology, specific polymerase chain reaction, and/or spoligotyping. PPD, T-SPOT and acid-fast bacilli staining were used for diagnosis of TB. PPD positive was defined as the diameter of > 5 mm after 48–72 h.

BCG infection was classified in three groups according to the affected organs and location: (I) local BCG infection: lymphadenitis near the injection site; (II) regional BCG infection: regional lymphadenopathy or other lesions beyond the vaccination site (e.g., ipsilateral axillary, supraclavicular, cervical lymph nodes); and (III) disseminated BCG infection: infections in remote sites or proved by blood or bone marrow culture.

The results of G ((1–3)-β-d-glucan) tests and GM (galactomannan) tests were used as evidences for fungal disease. GM index was defined as positive when > 0.5. G index was defined as positive when > 100 pg/ml^8^. GM test was highly sensitive to the infection of Aspergillus^2^.

### Imaging modalities

All CT studies were performed with conventional techniques on a 64-slice CT (GE Healthcare, Princeton, NJ). Chest protocol included the following parameters: tube current 80–100 mAs, tube voltage 80–100 kV and gantry rotation time 350 ms, a matrix of 512 × 512, reconstructed width 0.625 mm. Omnipaque (iodine content 300 mg/mL) was injected at the dose of 1.5–2 ml/kg by power injector at the rate of 0.8–1 ml/s. Reconstructions were made in the coronal and sagittal planes.

Two experienced radiologists analyzed the CT findings. If there were discrepant readings, they discussed and got the common decision. The observers evaluated: consolidation, nodules, ground-glass opacity, mass, abscess, cavity, tree-in bud opacities, interlobular septal thickening, pulmonary scarring, bronchiectasis, emphysema, pleural thickening, mediastinal or hilar lymphadenopathy, axillary lymphadenopathy, chest wall invasion, calcification in the mediastinal or hilar lymph nodes and pulmonary parenchyma. The radiographic definitions were defined by the Fleischner Society nomenclature^15^. Consolidation appeared as a homogeneous increase lesion of lung. A nodule was defined as a rounded opacity, well or poorly defined, up to 3 cm in diameter while a mass more than 3 cm in diameter. Ground-glass opacity was hazy increased opacity in pulmonary parenchyma, with bronchial and vascular margins visible inside. Bronchiectasis was defined as localized or diffuse dilatation of a bronchus. A cavity presented as a gas-filled space, within pulmonary consolidation, a mass, or a nodule. Emphysema was a focal area of low attenuation, usually without visible walls. Mediastinal or hilar lymphadenopathy was defined by the short-axis diameter of the lymph nodes more than 10 mm.

### Statistical analysis

Data were presented in a descriptive way. Continuous parameters were presented as mean ± standard deviation and inter-quartile range (IQR). Categorized data were expressed as number and percentages. Unpaired Student's *t* test was used to compare the age of patients in two groups. The degree of inter-observer agreement was evaluated by Fleiss’ κ values: κ < 0.40, poor agreement; 0.40 < κ < 0.75, fair to good agreement and 0.75 < κ < 1.00, excellent agreement.

### Ethics approval and consent to participate

Informed consent forms were signed by the parents and ethics approval was approved by the institutional review boards of the Children’s Hospital of Fudan University.

## Results

### Demographic and clinical features

**Figure 1:**
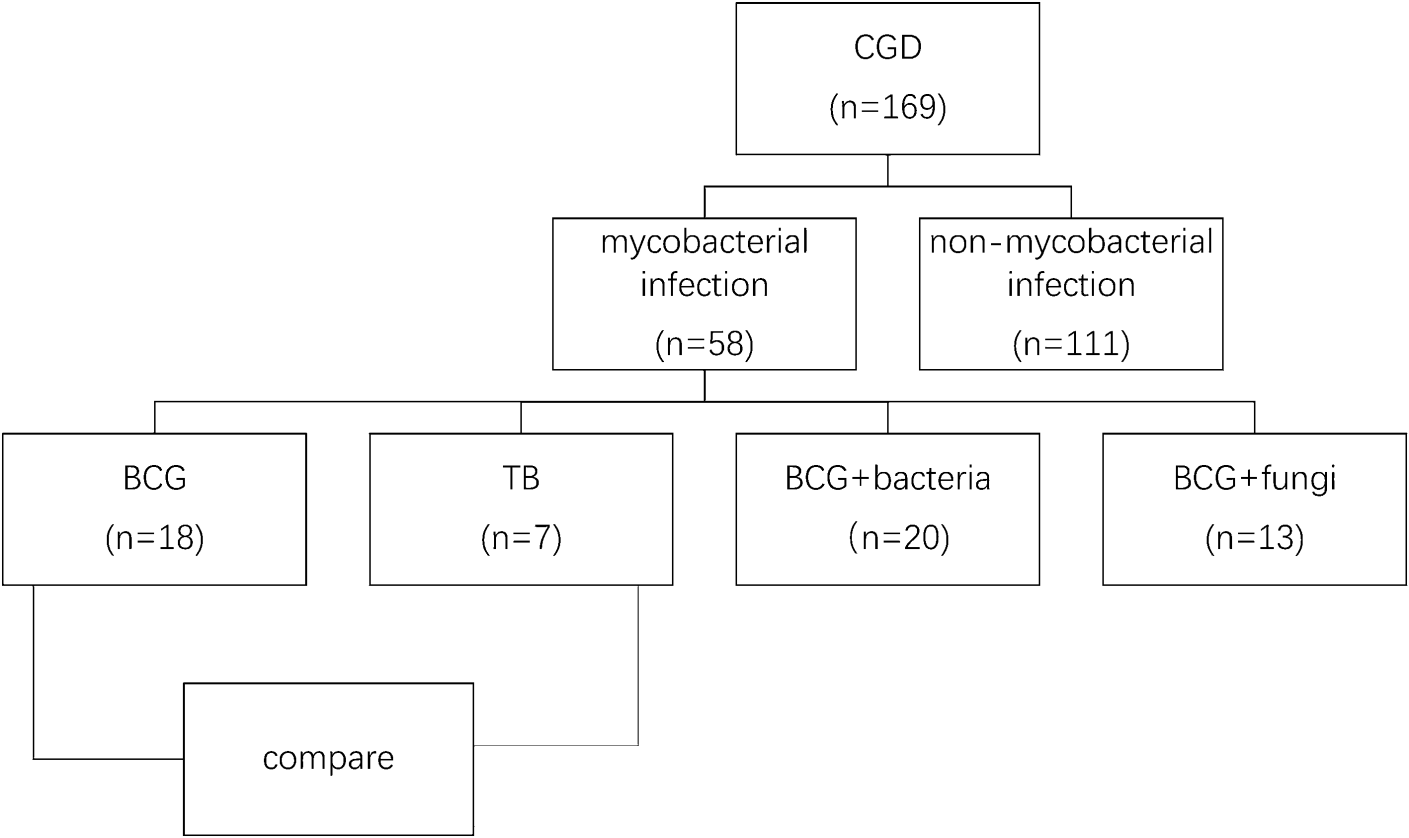
Study flowchart. *CGD* chronic granulomatous disease, *BCG* Bacillus Callmette–Güerin, *TB* tuberculosis.

The flow chart was listed in Fig. 1. Among all the 169 CGD patients in recent 20 years, 58 patients with pulmonary mycobacterial infection and chest CT examinations were included in this study. Fifty-seven (96.55%) cases were male, and 1 (3.44%) was female, indicating a high proportion of X-linked CGD. CYBB was identified as the main gene type. Of all the patients, 51 (87.93%) cases presented with BCG infection and 7 (12.07%) patients with TB. The average stimulation index was similar in both groups. The average age at BCG diagnosis was significantly lower than that of TB (6.33 ± 9.84 month-old vs. 58.14 ± 65.85 month-old, respectively, *p *< 0.05). In BCG group, apart from the patients with other bacterial (n = 20) and fungal (n = 13) infections, 18 patients presented solely with BCG disease. No other pathogens were found in TB group. The demographic and clinical features of subjects involved in the two groups were listed in Table 1.

**Table 1:** Distribution of genetic data and clinical infection.

**Sex**	
Male	57
Female	1
**Gene type**	
CYBB	53
CYBA	4
Unknown	1
**BCG**	58
Age at BCG diagnosis (month)	6.33 ± 9.84 (IQR, 2–12)
Stimulation index	5.13 ± 3.66
**TB**	
Age at TB diagnosis (month)	58.14 ± 65.85 (IQR, 29–89)
Stimulation index	5.87 ± 5.46
BCG only	18
TB only	7
BCG + bacteria	20
BCG + fungi	13

*BCG* Bacillus Callmette–Güerin, *TB* tuberculosis.

### BCG imaging findings

**Figure 2:**
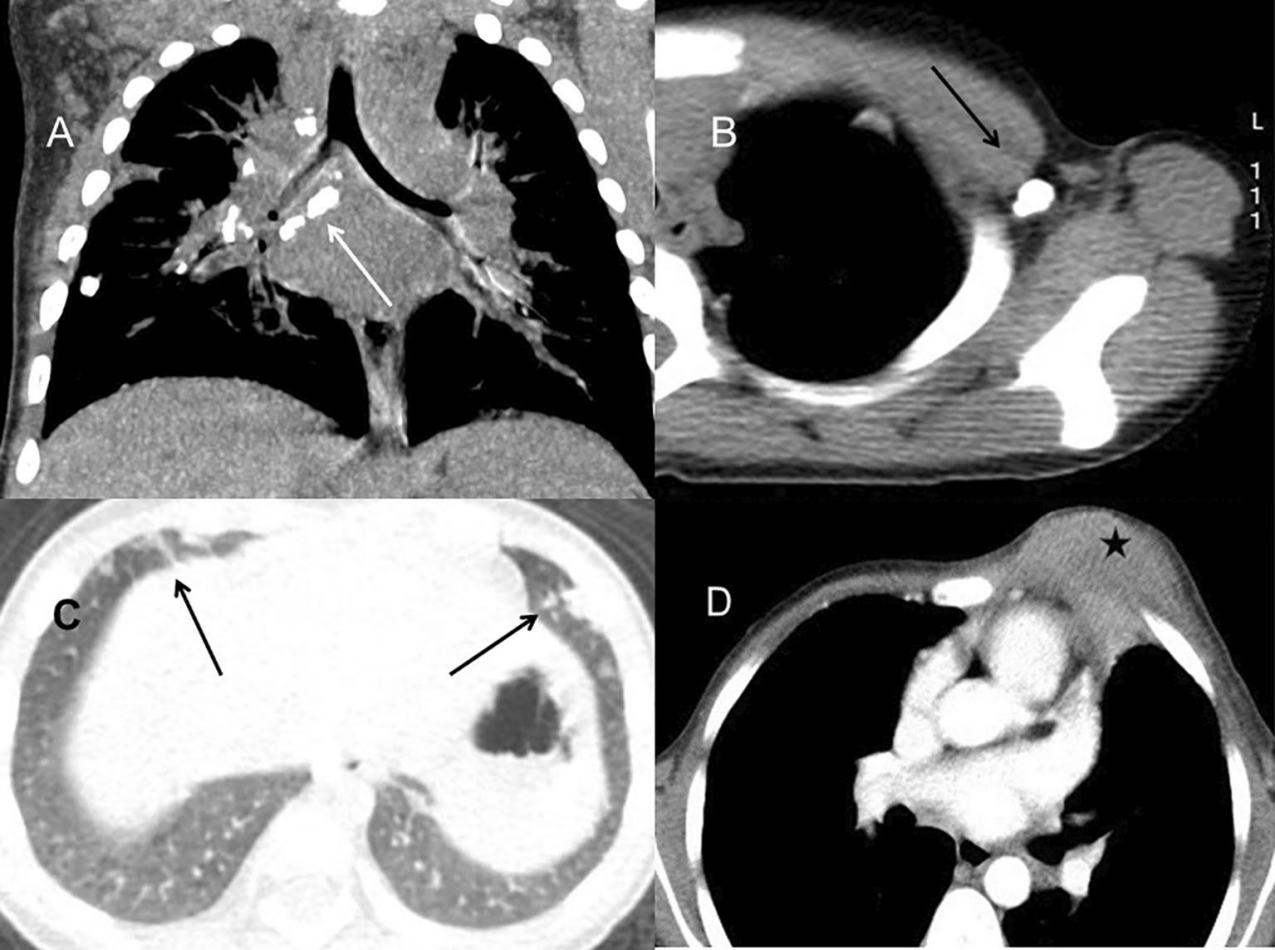
Pulmonary CT of BCG infection. (**A**) Coronal reformatted CT image of a 1-year-old boy demonstrated multiple nodular calcification within the mediastinal and hilar lymph nodes (long arrow). The calcified nodule in the right lower lobe was also found. (**B**) The left axillary calcification ipsilateral to the BCG injection site was found in a 1-year-old boy (long arrow). (**C**) Chest CT of a 9-month-old boy showed multiple pulmonary nodules in the bilateral lower lobe beneath the pleura (long arrow). (**D**) A 8-month-old boy with chest wall invasion. CT demonstrated a large chest wall mass with patchy areas of low density inside, suggesting the forming of abscess (star).

To avoid the disturbance of bacterial and fungal infection, we only included the 18 patients solely with BCG disease for imaging analysis. For those infected with only BCG (n = 18), local BCG infection lymphadenopathy was the first and most prevalent feature. The most common site was the mediastinal or hilar (n = 15) (Fig. 2A), the left axilla (n = 18) (Fig. 2B), and the ipsilateral cervical areas (n = 8). CT revealed a majority of hypodense lesions with slight enhancement. Four cases had abscess formation with ring enhancement and one was accompanied by fistula, presented as enhanced strips extending to the skin. Twelve had sand-like, streak-like or nodular calcification within the enlarged lymph nodes. Nine patients who had a follow-up CT scan after antituberculosis therapy (Isoniazid, rifampicin and ethambutol) were found with a decrease in the size of lymphadenopathy and an increase in calcification.

As disseminated BCG with CGD is extremely rare and the chest imaging data are limited, we further summarized the chest CT findings of 18 cases only with BCG disease in detail (Table 2). The presence of ground-glass opacities, multiple small nodules and short pulmonary scarring was most common among all the 108 pulmonary segments of 18 patients. The imaging showed small bilateral patchy ground-glass opacities without obvious propensity. 1–3 mm nodular lesions were mainly presented in the lower lobes beneath the pleura (Fig. 2C). Mild local pulmonary scarring was accompanied by slight tractive bronchiectasis and emphysema. Mass and patchy consolidation were found mostly in the bilateral upper lobes with multiple small cavities less than 3 mm in 2 cases. Small nodular calcification in the pulmonary parenchymal was identified in 20 segments of 7 patients without predominantly lobar location while one demonstrated chest wall invasion and surrounding soft-tissue mass (Fig. 2D), which was adjacent to the infectious mass in the left upper lobe.

**Table 2:** Number and location of main pulmonary CT manifestations in the 108 pulmonary segments of BCG infection (n = 18).

	RUL (%)	RML (%)	RLL (%)	LUL (%)	Lingula (%)	LLL (%)	N (%)
Consolidation	6 (5.56)	2 (1.85)	1 (0.93)	5 (4.63)	1 (0.93)	1 (0.93)	16 (14.81)
Nodules	3 (2.78)	3 (2.78)	7 (7.41)	2 (1.85)	4 (3.70)	9 (8.33)	28 (25.93)
Cavity	2 (1.85)	0	0	0	0	0	2 (1.85)
Ground-glass opacity	9 (8.33)	7 (7.41)	8 (44.44)	9 (8.33)	7 (7.41)	10 (9.26)	50 (46.30)
Tree-in bud opacities	1 (0.93)	2 (1.85)	0	0	0	0	3 (2.78)
Bronchiectasis	3 (2.78)	0	1 (0.93)	2 (1.85)	0	1 (0.93)	7 (6.48)
Emphysema	6 (5.56)	2 (1.85)	5 (4.63)	4 (3.70)	2 (1.85)	6 (5.56)	25 (23.15)
Pulmonary scarring	10 (9.26)	5 (4.63)	7 (7.41)	6 (5.56)	5 (4.63)	7 (7.41)	40 (37.04)
Mass	3 (2.78)	1 (0.93)	0	0	2 (1.85)	2 (1.85)	8 (7.41)
Interlobular septal thickening	1 (0.93)	2 (1.85)	3 (2.78)	0	0	3 (2.78)	9 (8.33)
Pulmonary calcification	3 (2.78)	3 (2.78)	4 (3.70)	3 (2.78)	2 (1.85)	5 (4.63)	20 (18.52)

*RUL* right upper lobe, *RML* right middle lobe, *RLL* right lower lobe, *LUL* left upper lobe, *LLL* left lower lobe, *Lingula* left lingula lobe.

**Figure 3:**
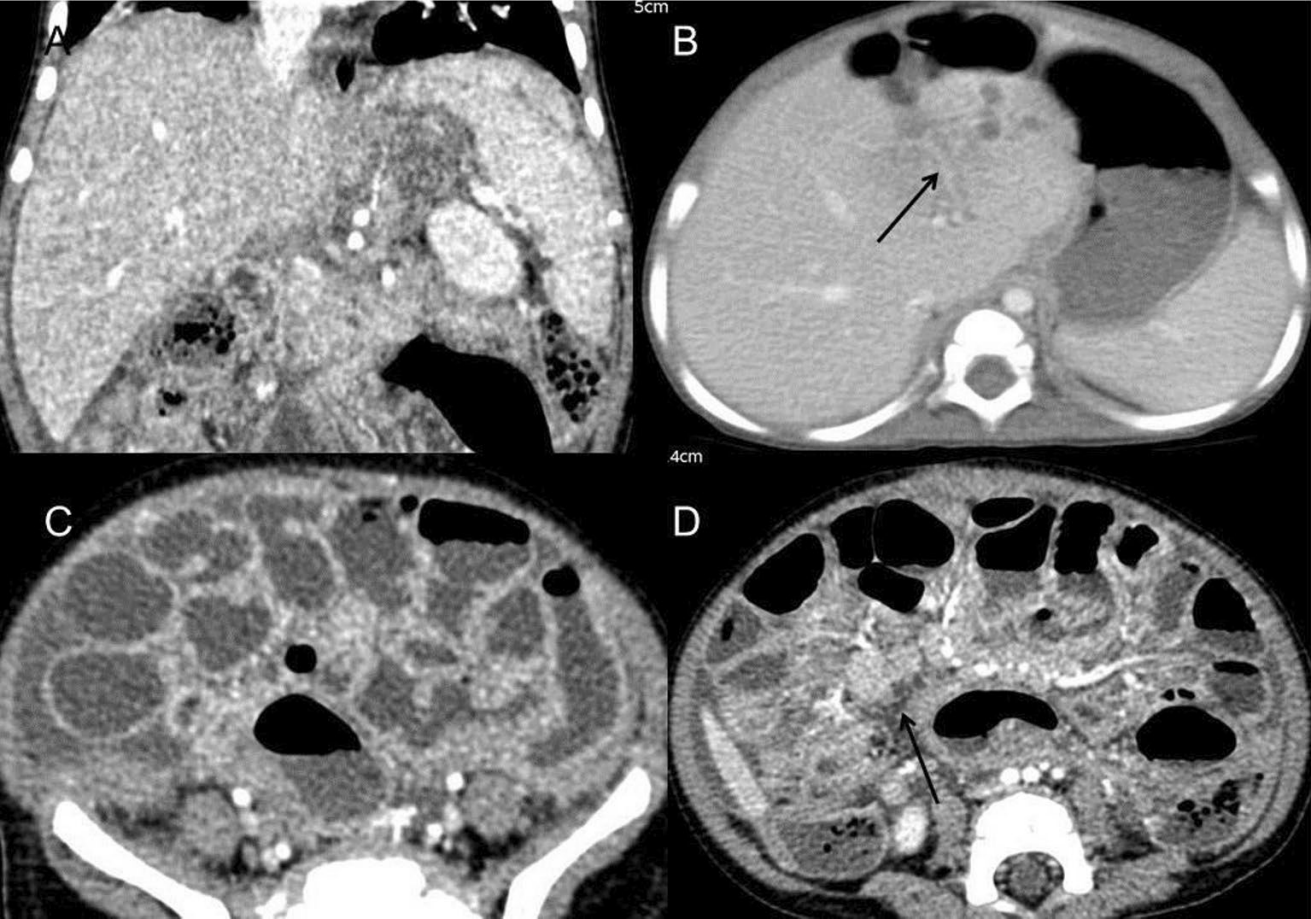
Abdominal visceral involvements of BCG infection. (**A**) Coronal post-contrast CT showed hepatosplenomegaly with homogeneous enhancement in a 1-year-old boy. (**B**) A 8-month-old boy with hepatic abscess. CT scan demonstrated the presence of multiple low-attenuated lesions in the left lobe (long arrow). Most lesions were less than 5 mm and some with ring enhancement. Edema around the left portal vein were also noticed. (**C**,**D**) A 1-year-old boy with enteritis. The widely thickened intestinal wall and effusion within the lumen were found on abdominal CT. The small amount of ascites also accompanied. The enlargement of mesenteric lymph nodes was noticed (**D**, long arrow).

Other visceral involvement included hepatosplenomegaly (n = 5), abscesses in the liver (n = 3), calcaneus osteomyelitis (n = 1) and enteritis (n = 1) (Fig. 3). In patients with hepatic abscess, contrast-enhanced CT scanning demonstrated the presence of multiple nodules with a central, low-attenuated mass, most of which were less than 5 mm. Patients with enteritis showed markedly thickened and enhanced intestinal wall on abdominal CT, accompanied by a small amount of ascites. One child was detected with skeletal infection in the calcaneal. Multiple osteolytic lesions showed periosteal reaction and cortical disruption, all of which regressed following appropriate anti-tuberculosis treatment.

We also have reviewed the 13 patients with BCG and fungal infection (Aspergillus or Candida). The 13 patients all presented with multiple nodules and infiltrations in a random distribution and halo signs around nodules could be found in 3 patients. In 1 patient with Aspergillus infection, large cavities were found inside the mass without calcification or air crescent sign. Mild enlargement of mediastinal or hilar lymph nodes (10–15 mm) and slight fibrosis were also identified.

### TB imaging findings

**Figure 4:**
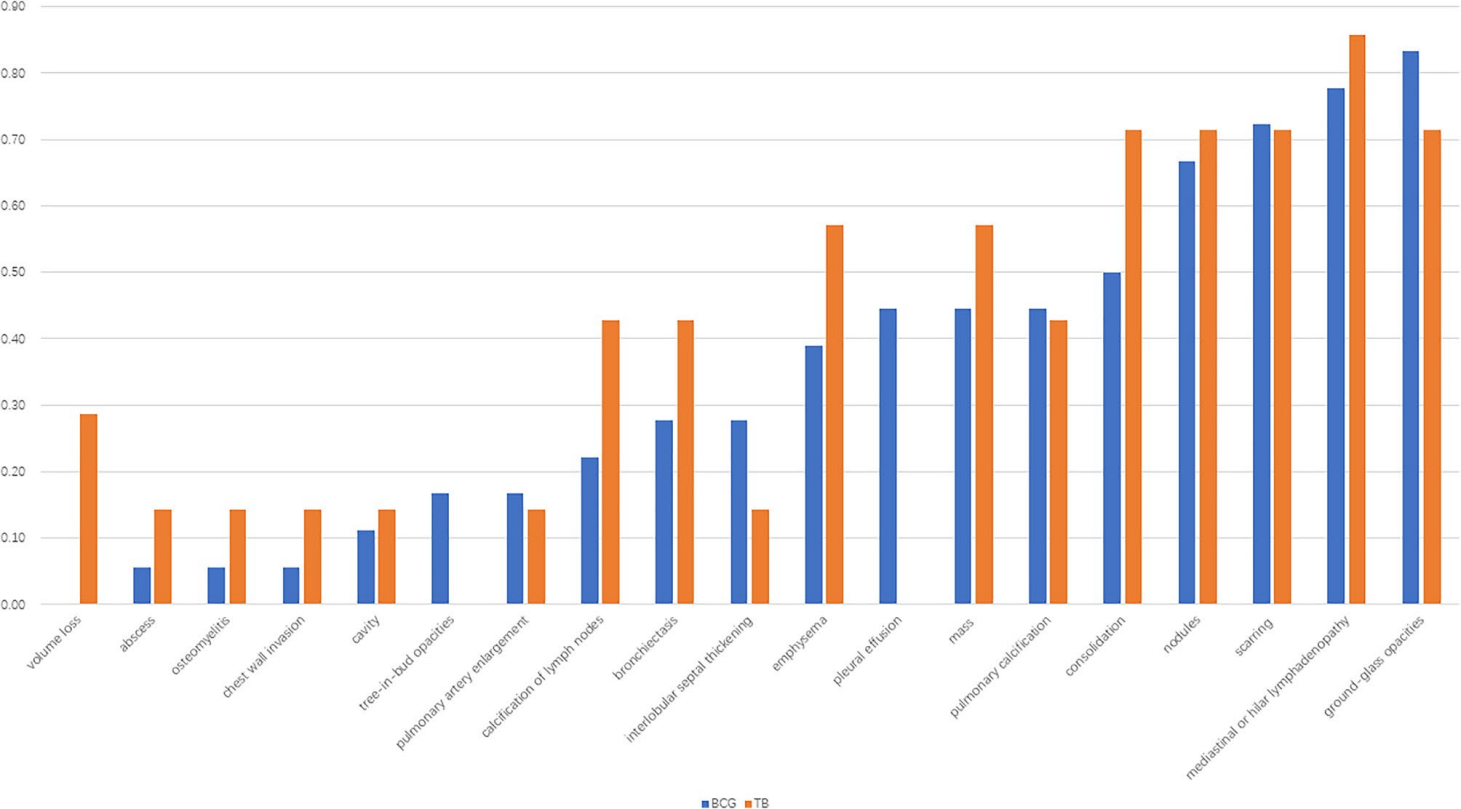
Percentage of thoracic lesions in patients with BCG and TB infections.

**Figure 5:**
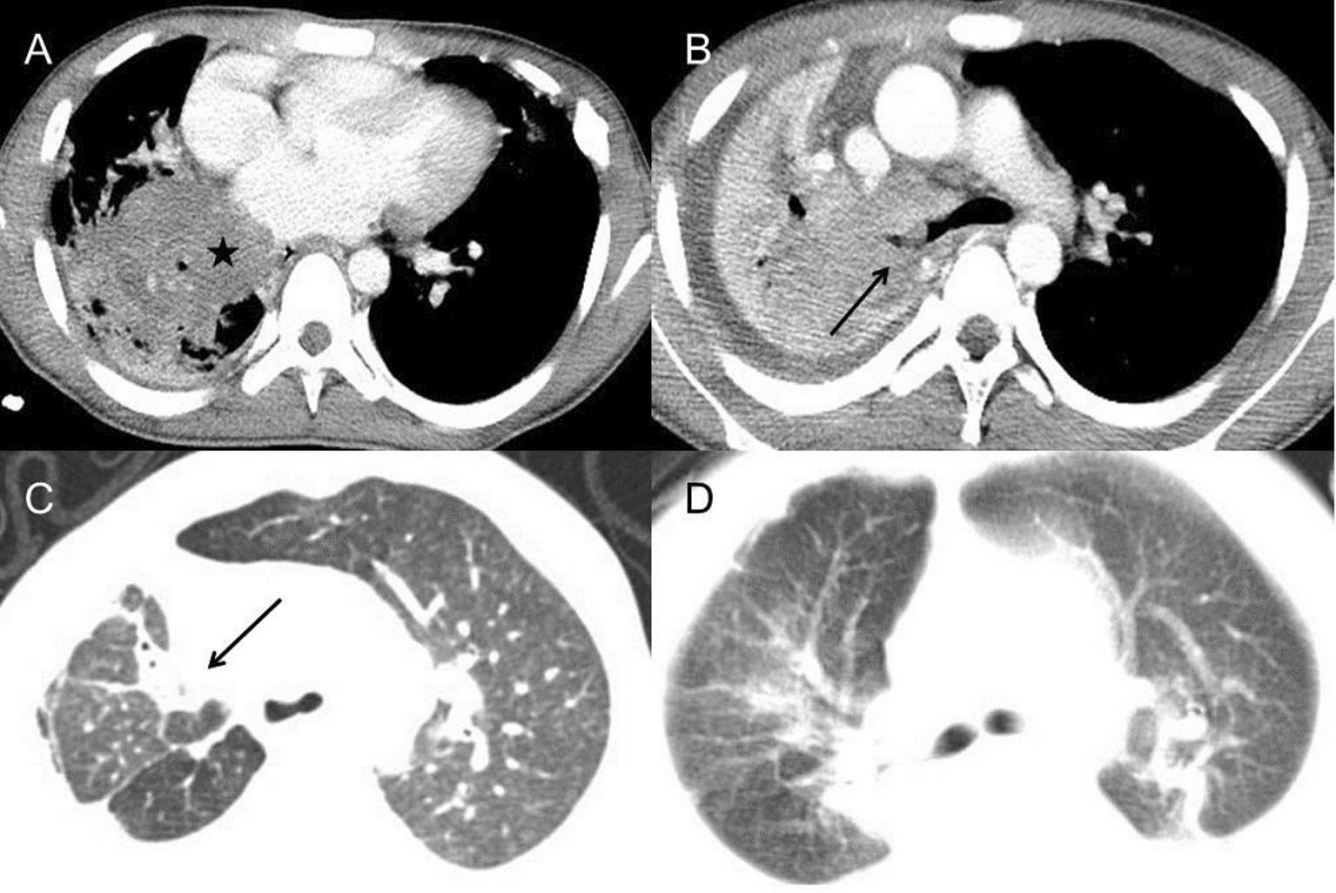
Pulmonary CT of TB infection. (**A**,**B**) 16-year-old boy with TB infection. (**A**) Post-contrast CT showed a large mass in the right lower lobe, about 5 cm in diameter (star). Multiple small cavities were found in the adjacent area of consolidation. (**B**) A nodule in the right bronchus led to atelectasis of the right lung, which was identified as TB granuloma formation by bronchoscopy (long arrow). The right pleural effusion was also found. (**C**) In a 3-year-old boy, axial CT scan showed scarring, segmental atelectasis and volume loss in the right upper lobe (long arrow). Slight bronchiectasis could be found inside the area of consolidation. (**D**) Segmental consolidation in bilateral upper lobes with adjacent ground-glass opacity were found in a 2-year-old boy.

The percentage of all thoracic lesions in patients with TB infections were compared with BCG in Fig. 4. Larger masses with heterogeneous enhancement and calcification were more common in TB infection with the biggest up to 5 cm in diameter (Fig. 5A). Six patients were found with more remarkable mediastinal or hilar lymphadenopathy with higher rate of calcification than BCG infection. Some lymph nodes fused into masses with caseous necrosis inside and compressed the trachea. One patient was identified with TB granuloma of the bronchial mucosa under bronchoscope, leading to atelectasis of the right lung (Fig. 5B). CT showed severer bronchiectasis and emphysema in 5 (71.43%) patients due to the extensive pulmonary fibrosis. Two patients developed architectural distortion of the lung and pulmonary volume reduction, which could not be found in BCG infection (Fig. 5C). Large areas of consolidation were more common in TB than BCG infection while no tree-in-bud opacities were identified (Fig. 5D). Chest wall invasion was found in 1 patient accompanied with spinal osteomyelitis and paravertebral abscess.

**Figure 6:**
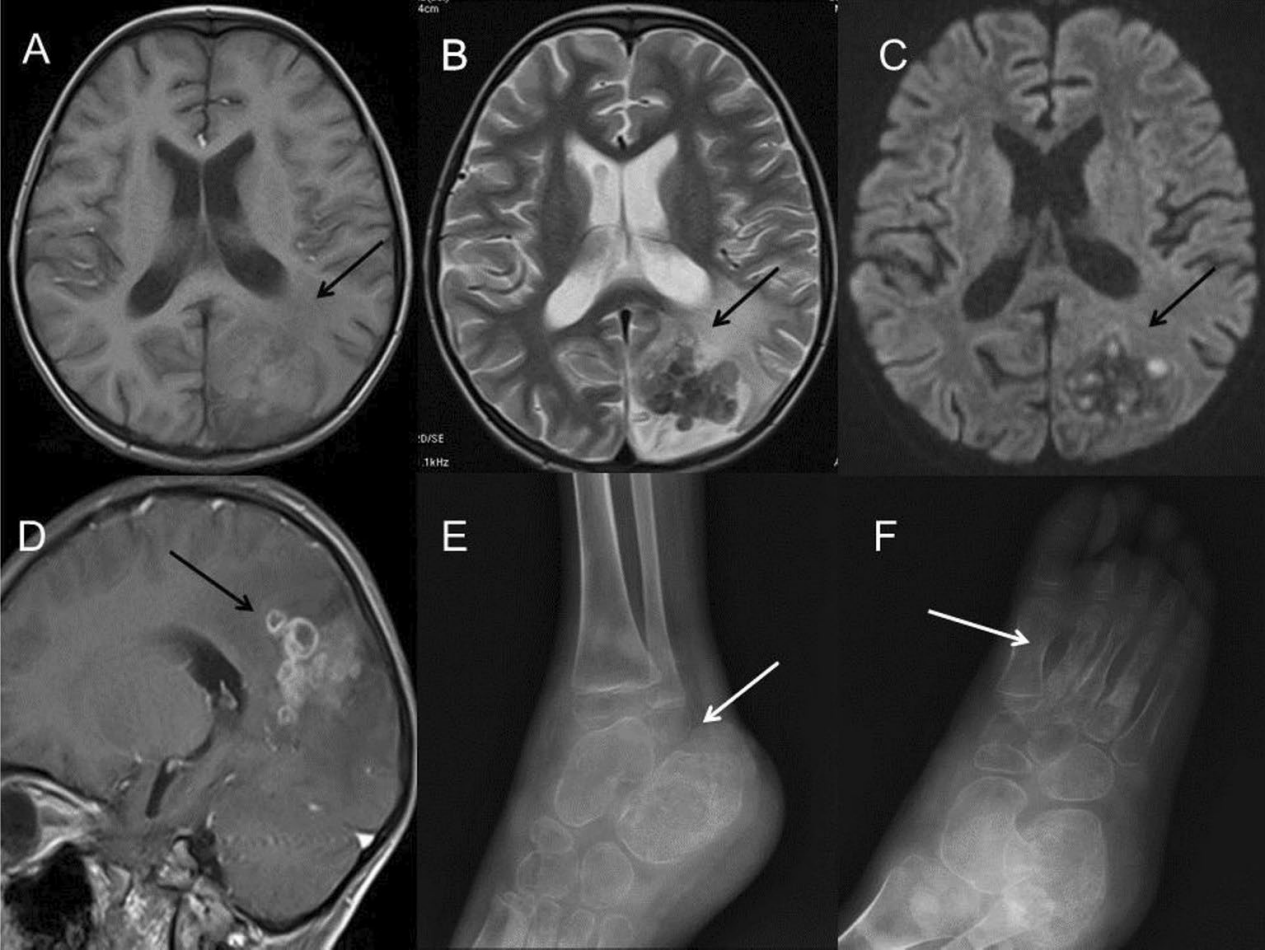
A 5-year-old boy with infection of TB. (**A**–**D**) MRI showed multiple small cerebral abscesses (long arrow) in the left parietal and occipital lobes, which had high intensity on T1WI (**A**), low intensity on T2WI (**B**), high intensity on DWI sequence (**C**) and annular enhancement on post-contrast T1WI (**D**), suggesting the presence of caseous necrosis. Extensive cerebral edema could be found around the lesions. (**E**,**F**) Osteomyelitis in the right tibia and foot was found in the same patient, demonstrating local bone erosions and osteosclerosis in the lower metaphysis of the tibia, the calcaneal and the 2nd–4th metatarsal bones (long arrow). Periosteal thickening along the metatarsal bones and swelling soft tissue of the right ankle and foot were found.

The other involved sites included hepatosplenomegaly (n = 2), kidney abscess (n = 1), right tibiofibular bone and foot osteomyelitis (n = 1), cerebral abscess (n = 1) and enteritis (n = 1) (Fig. 6). The imaging features of enteritis were similar to BCG infection, including extensive intestinal dilation and thickened intestinal wall on CT. One patient had multiple small cerebral abscesses in the left parietal and occipital lobes, showing high signal on T1WI and DWI, low signal on T2WI sequence and annular enhancement, indicating the possibility of caseous necrosis. The patient also had osteomyelitis in the right tibiofibular joint and foot, demonstrating local bone erosions and swelling of the surrounding soft tissue. All patients were treated with multiple-drug therapy (Isoniazid, rifampicin and ethambutol) and INF-therapy.

### Inter-observer correlation

Agreement between the two radiologists was strong for determining the main chest CT features. For the diagnosis of pathogens, the agreement of fungal infections (κ value of 0.89) and BCG infection (κ value of 0.76) was good. There was moderate agreement between TB infections (κ value of 0.52), and weak agreement in the diagnosis of bacterial infections (κ value of 0.38).

## Discussion

BCG vaccine has existed for 80 years and has been proved highly useful in preventing severe TB infections. In China, neonatal BCG vaccination has become a routine as suggested by the World Health Organization. According to the literature, the incidence of BCG complication is less than 1% in immunocompetent hosts, most of which are presented as local lymph nodes diseases^5,6^. However, there are concerns about the safety of BCG among PID patients including CGD. Deffert reported that in 297 cases of CGD patients with mycobacterial infections, BCG was most commonly identified in 220 (74%) patients, while TB was reported in only 59 (20%) patients^16^. Francesca examined 71 patients with CGD from 20 countries and found 31 (44%) patients had TB, and 53 (75%) presented BCG infection^14^. In a review of 93 cases of CGD in Mexico, 54 (58%) cases developed BCG infection after vaccine while TB occurred in 27 (29%) patients^7^. The BCG infection usually occurs in the first decade of life, especially within 1 year after vaccination, much earlier than the onset of TB^17,18^. The finding was consistent with previous studies. In the research, BCG has a high incidence of complication and earlier onset than TB, with the earliest presentation of BCG disease 1 month after birth.

The impairment in generation of reactive oxygen species would cause the infection to spread in the lymphatic and the blood system, with the lymphatic system being most vulnerable to infections^12,19^. In the cohort, lymphadenopathy, whether regional or disseminated, was the most common feature. BCG in China is administered in the left arm routinely. The most common location was in the left axillary ipsilateral to the injection site, followed by the ipsilateral cervical areas that develop along the lymphatic system^20^. The central abscess and rim enhancement could be regarded as a sign of progress, indicating caseous necrosis. After treatment, serial CT studies have been used to document the resolution of BCG-related lymphadenopathy, including changes in size and calcification, which are similar to the lymphadenopathy of tuberculosis.

Patients with PID usually have more severe BCG infection than patients without PID. Disseminated BCG involves multiple systems with fatal consequences in most cases^18^. The incidence is estimated to be 0.59 per 1 million and almost occurs in children with immunodeficiency disorders^21,22^. The lung has been proved to be the most common site and may be the first involved visceral organ. However, the lack of typical imaging characteristics could lead to inaccurate diagnosis^23–25^. Nodules and exudation have been reported as the most common manifestations, and some are characterized by small nodular calcification in the pulmonary parenchyma and lymphoid nodes^26^. In the cohort, pulmonary lesions presented multiple manifestations, including granulomas forming pathologically characterized by multiple nodules and masses mainly in the bilateral lower lobes, bearing similarities with the infection of fungus like aspergillus and candida, while the incidence of calcification is relatively lower in the fungal infection. The left axillary lymphadenopathy also highly suggests the existence of BCG infection. The regions of ground-grass opacity and consolidation vary from subtle patches to bilateral multi-segment distribution. Other lesions like interstitial pneumonia and fibrotic changes were local and mild without obvious volume loss.

In TB-endemic countries, children with CGD are at high risk of infection. In Argentina, Hong Kong, and Iran, up to 11%, 54.5%, and 31.7% of patients with CGD were found to have TB infection respectively^26^. We also observed a high incidence of TB in our cohort, most of which presented more severe infections than BCG group. Radiologic manifestations varied including heterogeneous patchy of consolidation, large masses with cavitation, multiple nodules, and obvious lymphadenopathy. Miliary lesion has not been found in our group. Progressive fibrosis with volume loss and tractive bronchiectasis in the upper lobes occurred in almost 1/3 of patients, much severe than BCG. Lymphadenopathy is another feature of TB, which larger in size and most seen in the bilateral hilar area. Compared with TB infection, BCG lymphadenitis is more generalized and dispersed. TB mostly affects the elderly, and children below 15 years old only constitute less than 5% of all cases^26–28^. In the paper, the BCG group has a much younger average age of onset than the TB group.

In CGD patients, mycobacterial infection also affects other visceral organs in a disseminated pattern, with liver and spleen as the most common infected organs^29^. Hepatosplenomegaly and multiple small abscesses have been observed, unlike the abscess of other pyogenic infection. Osteomyelitis, central nervous system infection and intestinal involvement have also been reported in the published literatures, but with relatively lower morbidity. The bone infection of BCG and TB has similar radiographic appearance characterized by osteolytic destruction and periosteal reaction, usually in the epiphysis and metaphysis of the long bone^30^. In the small or irregular bones, osteolytic destruction and dilation can be found with soft-tissue swelling^31,32^.

As an attenuated live vaccine, BCG presents lower pathogenicity and better response to anti-tuberculous treatment than TB. However, BCG has a higher incidence, and BCG disease might be the first sign of CGD caused by mandatory vaccination after birth, earlier than bacteria or fungi. Therefore, it is suggested that an evaluation of underlying immunodeficiency should be given for infant and children diagnosed as BCG disease^33^. In addition, for neonate with a family history of PIDs, BCG vaccination should be delayed until PID is excluded.

## Conclusion

In our cohort, the onset of BCG disease was much early than TB, which might be the first sign of PID. Lymphadenopathy, especially in the left axillary ipsilateral to the injection site and the ipsilateral cervical areas was the most common feature, which may regress and form calcification after treatment. The pulmonary manifestation of BCG infection was less remarkable than that of TB, but with higher incidence. TB infections in CGD patients usually bear serious consequences (fibrosis with volume loss and tractive bronchiectasis). The involvements of other systems were also common. For CGD patients, radiologists should be alert to the imaging features for early recognition of mycobacterial infections.

## Data Availability

The datasets used and/or analysed during the current study available from the corresponding author on reasonable request.

## Abbreviations

CGD: Chronic granulomatous disease
PID: Primary immunodeficiency disease
NADPH: Nicotinamide adenine dinucleotide phosphate
TB: Tuberculosis
BCG: Bacillus Callmette–Güerin

## References

[1] Matute JD (2009). A new genetic subgroup of chronic granulomatous disease with autosomal recessive mutations in P40 Phox and selective defects in neutrophil NADPH oxidase activity. Blood.

[2] Zhou Q (2018). A cohort of 169 chronic granulomatous disease patients exposed to BCG vaccination: A retrospective study from a single center in Shanghai, China (2004–2017). J. Clin. Immunol..

[3] Jabado N (1998). Invasive pulmonary infection due to *Scedosporium apiospermum* in two children with chronic granulomatous disease. Clin. Infect. Dis..

[4] Tao NN (2019). Epidemiological characteristics of pulmonary tuberculosis among children in Shandong, China, 2005–2017. BMC Infect. Dis..

[5] Zwerling A (2011). The BCG World Atlas: A database of global BCG vaccination policies and practices. PLoS Med..

[6] Krysztopa-Grzybowska K, Paradowska-Stankiewicz I, Lutyńska A (2012). The rate of adverse events following BCG vaccination in Poland. Przegl Epidemiol..

[7] Blancas-Galicia L (2020). Genetic, immunological, and clinical features of the first Mexican cohort of patients with chronic granulomatous disease. J. Clin. Immunol..

[8] Li T (2019). Genetic and clinical profiles of disseminated *Bacillus* Calmette–Guérin disease and chronic granulomatous disease in China. Front. Immunol..

[9] Sadeghi-Shanbestari M (2009). Immunologic aspects of patients with disseminated Bacille Calmette–Guerin disease in North-West of Iran. Ital. J. Pediatr..

[10] Winkelstein JA (2000). Chronic granulomatous disease. Report on a national registry of 368 patients. Medicine (Baltimore).

[11] Ying W (2014). Clinical characteristics and immunogenetics of BCGosis/BCGitis in Chinese children: A 6 year follow-up study. PLoS One.

[12] Marciano BE (2014). BCG vaccination in patients with severe combined immunodeficiency: Complications, risks, and vaccination policies. J. Allergy Clin. Immunol..

[13] Mahdaviani SA (2014). Pulmonary computed tomography scan findings in chronic granulomatous disease. Allergol. Immunopathol. (Madr)..

[14] Conti F (2016). Mycobacterial disease in patients with chronic granulomatous disease: A retrospective analysis of 71 cases. J. Allergy Clin. Immunol..

[15] Hansell DM (2008). Fleischner Society: Glossary of terms for thoracic imaging. Radiology.

[16] Deffert C (2014). Bacillus Calmette–Guerin infection in NADPH oxidase deficiency: Defective mycobacterial sequestration and granuloma formation. PLoS Pathog..

[17] Rezai MS, Khotaei G, Mamishi S, Kheirkhah M, Parvaneh N (2008). Disseminated Bacillus Calmette–Guerin infection after BCG vaccination. J. Trop. Pediatr..

[18] Afshar PS, Siadati A, Mamishi S, Tabatabaie P, Khotaee G (2006). Disseminated *Mycobacterium*
*bovis* infection after BCG vaccination. Iran. J. Allergy Asthma Immunol..

[19] Yang CS, Yuk JM, Jo EK (2009). The role of nitric oxide in mycobacterial infections. Immune Netw..

[20] Rigouts L (2009). Clinical practice: Diagnosis of childhood tuberculosis. Eur. J. Pediatr..

[21] Roos D (2010). Hematologically important mutations: The autosomal recessive forms of chronic granulomatous disease (second update). Blood Cells Mol. Dis..

[22] Köker MY (2013). Clinical, functional, and genetic characterization of chronic granulomatous disease in 89 Turkish patients. J. Allergy Clin. Immunol..

[23] Shrot S, Barkai G, Ben-Shlush A, Soudack M (2016). BCGitis and BCGosis in children with primary immunodeficiency—Imaging characteristics. Pediatr. Radiol..

[24] Kido J, Mizukami T, Ohara O, Takada H, Yanai M (2015). Idiopathic disseminated Bacillus Calmette–Guerin infection in three infants. Pediatr. Int..

[25] Kawashima H (2007). Hazards of early BCG vaccination: BCGitis in a patient with chronic granulomatous disease. Pediatr. Int..

[26] Lee PP (2008). Susceptibility to mycobacterial infections in children with X-linked chronic granulomatous disease: A review of 17 patients living in a region endemic for tuberculosis. Pediatr. Infect. Dis. J..

[27] Howie S (2005). Tuberculosis in New Zealand, 1992–2001: A resurgence. Arch. Dis. Child..

[28] Lamb, G. S. & Starke, J. R. Tuberculosis in infants and children. *Microbiol. Spectr*. **5** (2017). 10.1128/microbiolspec.tnmi7-0037-2016 PMC11687478 28387193

[29] Andronikou S, Wieselthaler N (2004). Modern imaging of tuberculosis in children: Thoracic, central nervous system and abdominal tuberculosis. Pediatr. Radiol..

[30] Hugosson C, Harfi H (1991). Disseminated BCG-osteomyelitis in congenital immunodeficiency. Pediatr. Radiol..

[31] Teo HE, Peh WC (2004). Skeletal tuberculosis in children. Pediatr. Radiol..

[32] Han TI, Kim IO, Kim WS, Yeon KM (2000). Disseminated BCG infection in a patient with severe combined immunodeficiency. Korean J. Radiol..

[33] Boisson-Dupuis S (2015). Inherited and acquired immunodeficiencies underlying tuberculosis in childhood. Immunol. Rev..

